# Low forced vital capacity predicts poor prognosis in gastric cancer patients

**DOI:** 10.18632/oncotarget.15953

**Published:** 2017-03-07

**Authors:** Fan Feng, Yangzi Tian, Yuan Zang, Li Sun, Liu Hong, Jianjun Yang, Man Guo, Xiao Lian, Daiming Fan, Hongwei Zhang

**Affiliations:** ^1^ Division of Digestive Surgery, Xijing Hospital of Digestive Disease, Fourth Military Medical University, 710032, Xi’an, Shaanxi, China; ^2^ Department of Dermatology, Xijing Hospital, Fourth Military Medical University, 710032, Xi’an, Shaanxi, China; ^3^ Department of Orthopedics, Xijing Hospital, Fourth Military Medical University, 710032, Xi’an, Shaanxi, China

**Keywords:** gastric cancer, forced vital capacity, maximal voluntary ventilation, postoperative complications, prognosis

## Abstract

Preoperative pulmonary function assessment is used to select surgical candidates and predict the occurrence of postoperative complications. The present study enrolled 1210 gastric cancer patients (949 males and 261 females). Forced vital capacity (FVC) and maximal voluntary ventilation (MVV) were measured as a percent of predicted values. We then analyzed associations between patient pulmonary function and both prognosis and postoperative complications. Patient 1-, 3- and 5-year overall survival rates were 88.8%, 65.7% and 53.0%, respectively. FVC and MVV optimal cutoff values were 87.0 (P=0.003) and 83.6 (P=0.026), respectively. Low FVC and low MVV were associated with higher rates of postoperative fever (23.8% vs. 13.9%, P<0.001; 17.8% vs. 13.3%, P=0.049, respectively) and poor patient prognosis (5-year overall survival: 43.5% vs. 57.6%, P=0.003; 51.8% vs. 54.3%, P=0.026, respectively). Only low FVC was an independent prognostic predictor for gastric cancer (P=0.012). In subgroup analyses, FVC was not associated with stage I or II gastric cancer patient prognoses (P>0.05), but low FVC was an independent risk factor for poor prognosis in stage III gastric cancer cases (P=0.004). These findings indicate that low FVC is predictive of poorer prognosis and higher risk of postoperative fever in gastric cancer patients.

## INTRODUCTION

Gastric cancer is the fifth most common malignancy, and the third leading cause of cancer-related death worldwide [1], although incidences have declined in recent decades. Surgical therapy remains the optimal treatment for non-metastatic gastric cancer. Still, even with advances in surgical techniques and adjuvant therapy options, advanced gastric cancer patient prognoses are poor [2].

Surgeons commonly encounter patients with impaired pulmonary function during preoperative evaluation. Pulmonary comorbidity increases the risk of postoperative respiratory complications [3]. Thus, preoperative evaluation of pulmonary function is widely used to select surgical candidates and predict the occurrence of postoperative respiratory complications, especially in the field of thoracic surgery [4]. Recent studies also investigated the influence of pulmonary function on abdominal surgery outcomes [5, 6]. However, the prognostic value of preoperative pulmonary function in gastric cancer patients has not yet been investigated. The present study assessed the value of pulmonary function in predicting gastric cancer patient prognosis and the likelihood of postoperative complications.

## RESULTS

**Figure 1:**
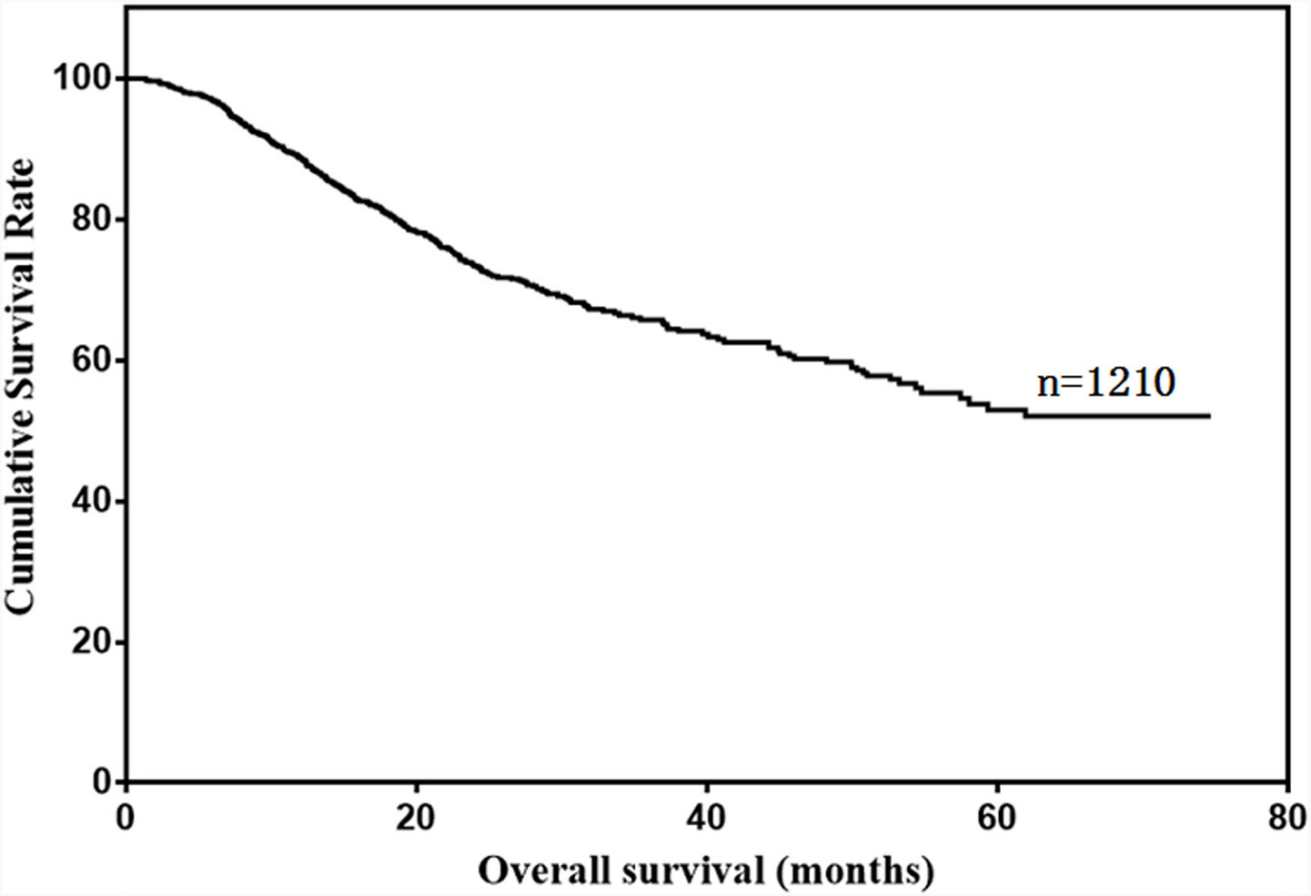
Overall survival of gastric cancer patients

Our study included 949 male (78.4%) and 261 female (21.6%) gastric cancer patients (Table 1). Median patient age was 59 years (range: 20–87), and median follow-up time was 25 months (range: 1–75). Patient 1-, 3- and 5-year overall survival rates were 88.8%, 65.7% and 53.0%, respectively (Figure 1).

**Table 1:** Clinicopathological characteristics of gastric cancer patients

Characteristics	Number (n=1210)	Percent
Gender		
Male	949	78.4
Female	261	21.6
Age		
≤60	701	38.0
>60	509	62.0
BMI		
<18.5	107	8.9
≥18.5-<25.0	862	71.2
≥25.0	241	19.9
Total protein		
<65.0	338	27.9
≥65.0	872	72.1
Albumin		
<40.0	264	21.8
≥40.0	946	78.2
Tumor location		
Upper third	425	35.1
Middle third	201	16.6
Lower third	502	41.5
Entire	82	6.8
Tumor size (cm)		
≤5	810	66.9
>5	400	33.1
Borrmann type		
I	155	15.7
II	320	32.3
III	426	43.1
IV	88	8.9
Pathological type		
Well differentiated	104	8.6
Moderately differentiated	308	25.5
Poorly differentiated	754	62.3
Signet ring cell or Mucinous	44	3.6
Tumor depth		
T1	223	18.4
T2	111	9.2
T3	433	35.8
T4	443	36.6
Lymph node metastasis		
N0	413	34.1
N1	210	17.4
N2	206	17.0
N3	381	31.5
Tumor stage		
I	265	21.9
II	330	27.3
III	615	50.8

Forced vital capacity (FVC) and maximal voluntary ventilation (MVV) optimal cutoff values were 87.0 (P=0.003) and 83.6 (P=0.026), respectively. Baseline characteristics of patients with low versus high FVC and MVV levels were analyzed and shown in Supplementary Table 1. We found that FVC level was associated with gender, age, body mass index (BMI), albumin, tumor size, and tumor stage (P<0.05). MVV level was associated with age, BMI, total protein, albumin, tumor size, lymph node metastasis, and tumor stage (P<0.05).

**Figure 2:**
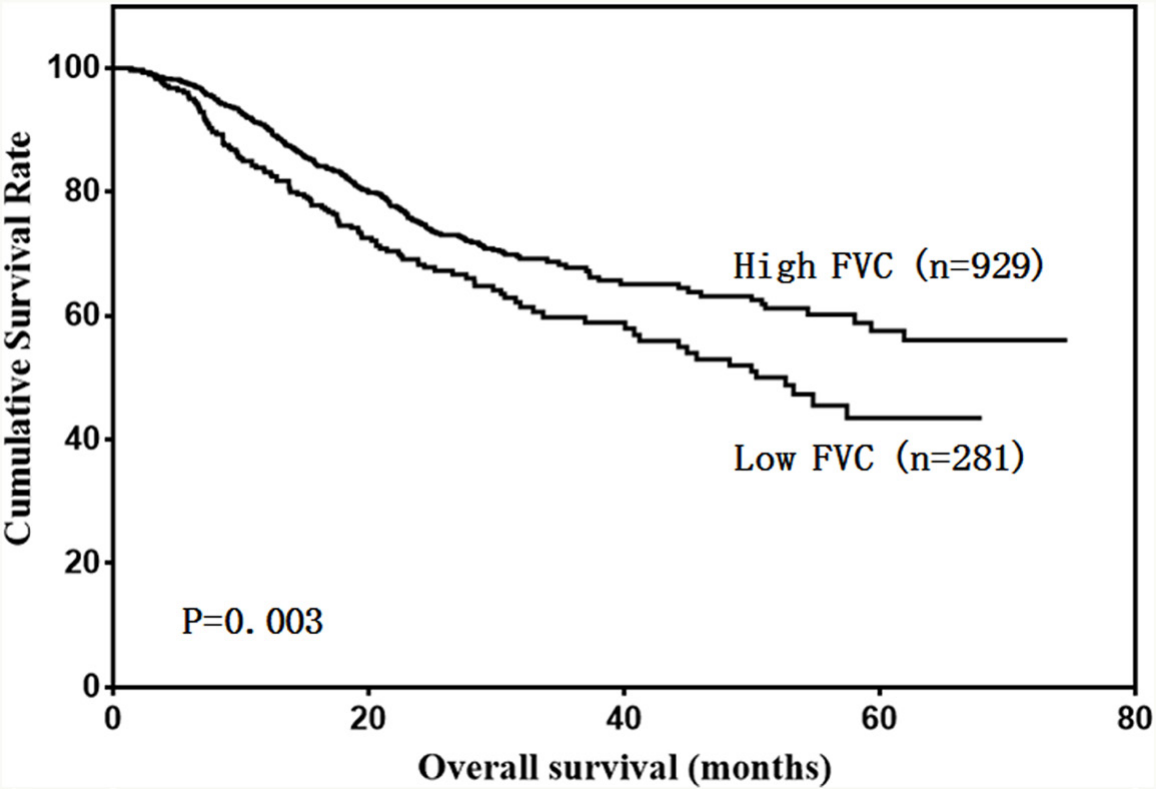
Patient overall survival according to FVC level

**Figure 3:**
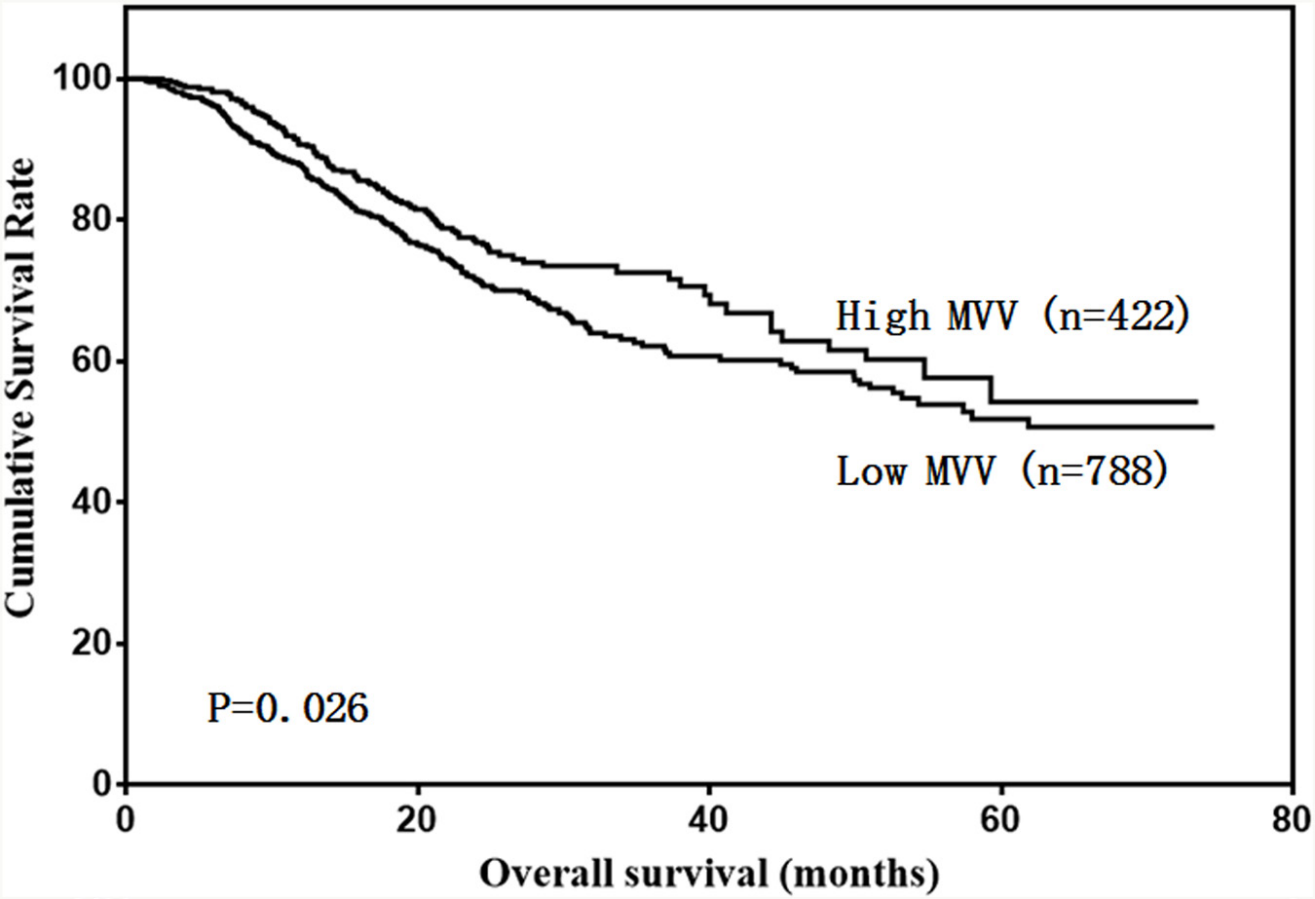
Patient overall survival according to MVV level

Our results showed that low FVC and low MVV were associated with poor prognosis in gastric cancer patients (Figure 2 & 3). A univariate analysis showed that patient age, BMI, total protein, albumin, tumor size, Borrmann type, pathological type, tumor depth, lymph node metastasis, tumor stage, FVC, and MVV were associated with prognosis (Table 2). However, only age, BMI, tumor depth, lymph node metastasis, and FVC were independent prognostic predictors (Table 3).

**Table 2:** Univariate analysis of risk factors for prognosis of gastric cancer

Prognostic factors	β	Hazard ratio (95% CI)	P value
Gender	−0.119	0.888(0.689-1.144)	0.357
Age	0.383	1.466(1.197-1.797)	0.000
BMI	−0.477	0.621(0.508-0.759)	0.000
Total protein	−0.249	0.780(0.629-0.967)	0.023
Albumin	−0.297	0.743(0.592-0.932)	0.010
Tumor location	0.023	1.023(0.923-1.134)	0.667
Tumor size	0.822	2.275(1.857-2.787)	0.000
Borrmann type	0.212	1.236(1.089-1.403)	0.001
Pathological type	0.535	1.707(1.453-2.005)	0.000
Tumor depth	0.941	2.562(2.206-2.977)	0.000
Lymph node metastasis	0.715	2.044(1.851-2.257)	0.000
Tumor stage	1.379	3.970(3.202-4.923)	0.000
FVC	−0.330	0.719(0.576-0.897)	0.003
MVV	−0.253	0.777(0.622-0.970)	0.026

**Table 3:** Multivariate analysis of risk factors for prognosis of gastric cancer

Prognostic factors	B	Hazard ratio (95% CI)	P value
Age	0.272	1.313(1.064-1.619)	0.011
BMI	−0.328	0.720(0.584-0.888)	0.002
Total protein	−0.022	0.979(0.742-1.290)	0.878
Albumin	−0.155	0.857(0.639-1.148)	0.300
Tumor size	0.201	1.222(0.992-1.507)	0.060
Borrmann type	0.096	1.100(0.976-1.240)	0.118
Pathological type	0.065	1.067(0.887-1.284)	0.494
Tumor depth	0.594	1.811(1.488-2.204)	0.000
Lymph node metastasis	0.481	1.618(1.445-1.811)	0.000
FVC	−0.296	0.743(0.590-0.937)	0.012
MVV	−0.097	0.908(0.719-1.146)	0.417

**Figure 4:**
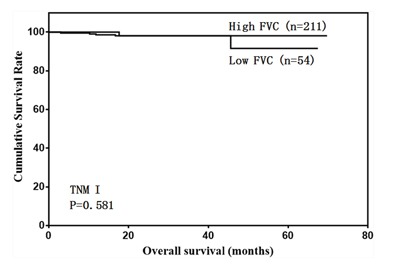
Overall survival of stage I patients according to FVC level

**Figure 5:**
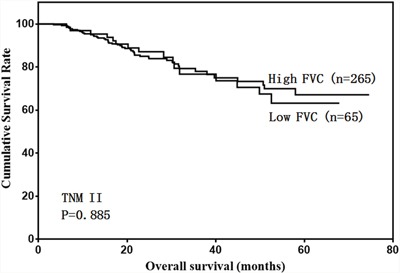
Overall survival of stage II patients according to FVC level

**Figure 6:**
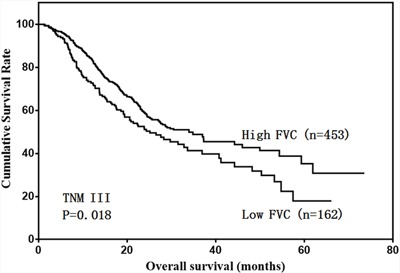
Overall survival of stage III patients according to FVC level

We then analyzed the predictive value of FVC in patients with different tumor stages. FVC was not associated with prognosis in stage I and II gastric cancer cases (Figure 4 & 5). However, low FVC was associated with poor prognosis in patients with stage III gastric cancer (Figure 6). Univariate and multivariate analyses showed that FVC was an independent risk factor for prognosis in stage III gastric cancer patients (Tables 4 & 5).

**Table 4:** Univariate analysis of risk factors for prognosis of stage III gastric cancer

Prognostic factors	β	Hazard ratio (95% CI)	P value
Gender	−0.031	0.969(0.734-1.280)	0.825
Age	0.234	1.264(1.008-1.585)	0.042
BMI	−0.290	0.748(0.595-0.941)	0.013
Total protein	−0.075	0.927(0.727-1.183)	0.544
Albumin	−0.168	0.846(0.653-1.096)	0.205
Tumor location	0.100	1.105(0.989-1.234)	0.077
Tumor size	0.215	1.240(0.989-1.555)	0.062
Borrmann type	0.122	1.130(0.988-1.292)	0.074
Pathological type	0.176	1.192(0.969-1.466)	0.096
Tumor depth	0.476	1.609(1.286-2.015)	0.000
Lymph node metastasis	0.519	1.680(1.372-2.057)	0.000
Lymphatic-vascular invasion	0.257	1.293(0.939-1.780)	0.116
Neural invasion	0.298	1.348(0.845-2.149)	0.210
FVC	−0.294	0.745(0.584-0.951)	0.018
MVV	−0.179	0.836(0.652-1.073)	0.160

**Table 5:** Multivariate analysis of risk factors for prognosis of stage III gastric cancer

Prognostic factors	β	Hazard ratio (95% CI)	P value
Age	0.228	1.256(0.999-1.577)	0.051
BMI	−0.089	0.915(0.878-0.953)	0.000
Tumor depth	0.625	1.869(1.489-2.346)	0.000
Lymph node metastasis	0.614	1.848(1.509-2.262)	0.000
FVC	−0.362	0.696(0.543-0.893)	0.004

Finally, we analyzed relationships between FVC and MVV levels and postoperative complications (Table 6). Low FVC and low MVV were associated with higher rates of postoperative fever (23.8% vs. 13.9%, P<0.001; 17.8% vs. 13.3%, P=0.049, respectively). In addition, low FVC was associated with a higher rate of wound infection (1.4% vs. 0.2%, P=0.029).

**Table 6:** Comparison of postoperative complications

Complications	FVC			MVV		
	<87.0n=281	≥87.0n=929	P value	<83.6n=788	≥83.6n=422	P value
Total cases	110	269	0.002	270	109	0.003
Fever	67	129	<0.001	140	56	0.049
Pneumonia	16	60	0.779	56	20	0.135
Wound infection	4	2	0.029	6	0	0.098
Wound disruption	8	8	0.017	11	5	1.000
Anastomosis leak	4	12	0.773	11	5	1.000
Abdominal bleeding	1	8	0.694	5	4	0.727
Chyle leakage	1	12	0.320	8	5	0.776
Pleural effusion	5	16	1.000	14	7	1.000
Gastric stasis	0	3	1.000	1	2	0.280
Ileus	4	18	0.799	17	5	0.266
Duodenal stump leak	0	1	1.000	1	0	1.000

## DISCUSSION

Pulmonary disease is seldom clinically diagnosed unless a patient presents with overt respiratory symptoms. Thus, preoperative screening for pulmonary disease usually depends on a given patient's previous medical history. Preoperative screening using pulmonary function testing is likely to be more valuable than conventional assessment in terms of evaluating pulmonary abnormalities and predicting postoperative complications [7]. However, while preoperative pulmonary function testing is accepted as an effective tool for predicting operative risk before thoracic surgery [8], it is not yet routinely performed for gastric cancer patients before surgery.

Associations between preoperative pulmonary function and postoperative pulmonary complications and patient mortality have been well investigated. However, data describing the impact of pulmonary disease on radical gastrectomy outcomes were controversial. Kim, *et al*. reported that pulmonary disease was associated with postoperative morbidity in a large, multicenter, laparoscopic gastrectomy study [9]. Jeong, *et al*. found that preoperative pulmonary function testing effectively predicted the risk of surgical complications and systemic complications in patients undergoing gastrectomy [10]. However, several other studies reported that pulmonary disease did not increase the risk of postoperative complications after gastric cancer surgery [11, 12]. The present study found that low FVC and low MVV were associated with higher incidence of postoperative fever.

The prognostic value of preoperative pulmonary function has mainly been investigated in thoracic surgery [13, 14]. Guo, *et al*. reported that FVC was an independent risk factor for the prognosis of non-small cell lung cancer patients who underwent curative resection, and FVC<80% predicted poor patient survival [13]. Matsuzaki, *et al*. associated low forced expiratory volume 1 (FEV1)/FVC ratios with reduced overall and disease-free survival in lung cancer patients undergoing thoracic surgery. The same group found that the carbon monoxide diffusing capacity of the lung and the inspiratory capacity/total lung capacity ratio were associated with patient prognosis [14]. To the best of our knowledge, no previous study has associated preoperative pulmonary function with gastric cancer patient prognosis. Our study associated low FVC and MVV with poor prognosis in gastric cancer patients, and FVC was an independent prognostic predictor.

Cachexia and weight loss in advanced gastric cancer patients were important factors predicting long-term survival. Poor respiratory function may be partly attributed to cancer-induced cachexia. Our study found that although BMI, total protein, and albumin were all associated with gastric cancer patient prognosis, FVC was the only independent risk factor for prognosis.

Multiple groups have investigated the association between FVC and survival in the general population [15–17]. Burnery, *et al*. reported that FVC, but not airway obstruction, predicts survival in asymptomatic adults without chronic respiratory diagnoses or persistent respiratory symptoms [16]. Low FVC was associated with increased mortality risk [18]. We suggest two possible explanations for these findings, both of which strengthen the case for using pulmonary function testing in gastric cancer patients prior to surgery. First, pulmonary function tests may reflect muscle strength and general energy levels, and physical and psychological disorders may manifest as lower values. Thus, these tests may indicate an individual patient's overall health. Second, poor fetal growth rates and lower birth weights may result in reduced lung function and increased risk of cardiovascular disease [19, 20]. In these cases, FVC may reflect overall cardiopulmonary function as well as general health.

There were several limitations in our present study. First, it was a retrospective analysis limited to a single center. Multi-center studies are needed to verify the predictive value of FVC. Second, our patient cohort was not large enough, and small sample sizes can result in biased statistical analyses. Third, we did not evaluate the predictive value of FVC after radical gastrectomy. Postoperative pulmonary function may play roles in gastric cancer patient prognosis, and should be explored.

Although preoperative pulmonary function has been associated with postoperative respiratory complications, the prognostic value of preoperative pulmonary function in gastric cancer patients undergoing radical surgery had not yet been investigated. In conclusion, our study demonstrated that low FVC and MVV were associated with poor prognosis and higher rates of postoperative fever in gastric cancer patients, and FVC was an independent prognostic predictor.

## MATERIALS AND METHODS

This study was performed at the Xijing Hospital of Digestive Diseases affiliated with the Fourth Military Medical University, China. From October 2008 to March 2015, a total of 1210 gastric cancer patients in our department were enrolled in the present study. Inclusion criteria were as follows: 1. without other malignant tumors, 2. without distant metastasis, 3. without neoadjuvant chemotherapy, 4. with radical D2 gastrectomy, 5. with preoperative pulmonary function test. This study was approved by the Ethics Committee of Xijing Hospital, and written informed consent was obtained from all patients before surgery.

All patients were treated with proximal, distal or total gastrectomy with D2 lymphadenectomy. The surgical procedure was based on the recommendations of the Japanese Gastric Cancer Treatment Guidelines [21]. Primary tumor depth and degree of lymph node involvement were defined according to the TNM classification. Postoperative chemotherapy was administrated according to the National Comprehensive Cancer Network guidelines.

Pulmonary function test was performed no more than seven days before surgery. FVC and MVV were measured by spirometry. Observed values were expressed as a percent of predicted values. Clinicopathological data, including gender, age, BMI, total protein, albumin, tumor location, tumor size, Borrmann type, type of resection, pathological type, tumor depth, lymph node metastasis and tumor stage, were collected. Postoperative complications, including fever, pneumonia, wound infection, wound disruption, anastomosis leakage, abdominal bleeding, chyle leakage, pleural effusion, gastric stasis, ileus and duodenal stump leakage, were also recorded. Patients were followed-up until November 2016, with enhanced chest and abdominal CT and gastroscopy every 3 months.

Data were processed using SPSS 22.0 for Windows (SPSS Inc., Chicago, IL, USA). Optimal FVC and MVV cutoff values for gastric cancer prognosis prediction were calculated using X-tile software [22]. Discrete variables were analyzed using Chi-square test or Fisher's exact test. Significant prognostic risk factors identified by univariate analysis were further assessed by multivariate analysis using the Cox's proportional hazards regression model. Overall survival was analyzed by Kaplan-Meier method. P≤0.05 was considered statistically significant.

## SUPPLEMENTARY MATERIALS FIGURES AND TABLES




