# Supplementary material for: Low forced vital capacity predicts poor prognosis in gastric cancer patients
Source: Oncotarget. 2017 Mar 7;8(17):28897–905. doi: 10.18632/oncotarget.15953 (PMC5438701; doi:10.18632/oncotarget.15953)
Supplement: Supplementary file 1 [file oncotarget-08-28897-s001.pdf]

Low forced vital capacity predicts poor prognosis in gastric cancer patients

SUPPLEMENTARY TABLE

Supplementary Table 1: Clinicopathological characteristics of gastric cancer patients

See Supplementary File 1
