# Supplementary material for: Low forced vital capacity predicts poor prognosis in gastric cancer patients
Source: Oncotarget. 2017 Mar 7;8(17):28897–905. doi: 10.18632/oncotarget.15953 (PMC5438701; doi:10.18632/oncotarget.15953)
Supplement: Supplementary file 2 [file oncotarget-08-28897-s002.docx]

Table S1 Clinicopathological characteristics of gastric cancer patients.

|  | FVC | |  | MVV | |  |
| --- | --- | --- | --- | --- | --- | --- |
| Characteristics | <87.0  n=281 | ≥87.0  n=929 | P value | <83.6  n=788 | ≥83.6  n=422 | P value |
| Gender |  |  | 0.000 |  |  | 0.186 |
| Male | 251 | 698 |  | 609 | 340 |  |
| Female | 30 | 231 |  | 179 | 82 |  |
| Age |  |  | 0.003 |  |  | 0.000 |
| ≤60 | 141 | 560 |  | 414 | 287 |  |
| >60 | 140 | 369 |  | 374 | 135 |  |
| BMI |  |  | 0.001 |  |  | 0.002 |
| <18.5 | 38 | 69 |  | 79 | 28 |  |
| ≥18.5-<25.0 | 177 | 685 |  | 573 | 289 |  |
| ≥25.0 | 66 | 175 |  | 136 | 105 |  |
| Total protein |  |  | 0.058 |  |  | 0.001 |
| <65.0 | 91 | 247 |  | 244 | 94 |  |
| ≥65.0 | 190 | 682 |  | 544 | 328 |  |
| Albumin |  |  | 0.000 |  |  | 0.002 |
| <40.0 | 88 | 176 |  | 193 | 71 |  |
| ≥40.0 | 193 | 753 |  | 595 | 351 |  |
| Tumor location |  |  | 0.986 |  |  | 0.607 |
| Upper third | 98 | 327 |  | 282 | 143 |  |
| Middle third | 48 | 153 |  | 135 | 66 |  |
| Lower third | 115 | 387 |  | 316 | 66 |  |
| Entire | 20 | 62 |  | 55 | 27 |  |
| Tumor size (cm) |  |  | 0.013 |  |  | 0.006 |
| ≤5 | 171 | 639 |  | 506 | 304 |  |
| >5 | 110 | 290 |  | 282 | 118 |  |
| Borrmann type |  |  |  |  |  | 0.645 |
| I | 32 | 123 | 0.059 | 97 | 58 |  |
| II | 63 | 257 |  | 214 | 106 |  |
| III | 118 | 308 |  | 289 | 137 |  |
| IV | 21 | 68 |  | 57 | 32 |  |
| Pathological type |  |  | 0.423 |  |  | 0.539 |
| Well differentiation | 24 | 80 |  | 68 | 36 |  |
| Moderately differentiation | 82 | 226 |  | 210 | 98 |  |
| Poorly differentiation | 166 | 588 |  | 480 | 274 |  |
| Signet ring cell or Mucinous | 9 | 25 |  | 30 | 14 |  |
| Tumor depth |  |  | 0.163 |  |  | 0.257 |
| T1 | 48 | 175 |  | 133 | 90 |  |
| T2 | 19 | 92 |  | 71 | 40 |  |
| T3 | 114 | 319 |  | 291 | 142 |  |
| T4 | 100 | 343 |  | 293 | 150 |  |
| Lymph node metastasis |  |  | 0.060 |  |  | 0.002 |
| N0 | 78 | 335 |  | 241 | 172 |  |
| N1 | 55 | 155 |  | 139 | 71 |  |
| N2 | 56 | 150 |  | 150 | 56 |  |
| N3 | 92 | 289 |  | 258 | 123 |  |
| Tumor stage |  |  | 0.032 |  |  | 0.009 |
| I | 54 | 211 |  | 157 | 108 |  |
| II | 65 | 265 |  | 206 | 124 |  |
| III | 162 | 453 |  | 425 | 190 |  |
